# Calculation of the Connected Dominating Set Considering Vertex Importance Metrics

**DOI:** 10.3390/e20020087

**Published:** 2018-01-28

**Authors:** Francisco Vazquez-Araujo, Adriana Dapena, María José Souto-Salorio, Paula M. Castro

**Affiliations:** 1 Department of Computer Engineering, Universidade da Coruña, Campus de Elviña, 15071 A Coruña, Spain; 2 Department of Computation, Universidade da Coruña, Campus de Elviña, 15071 A Coruña, Spain

**Keywords:** connected dominating set, complex networks, graph entropy, vertex importance

## Abstract

The computation of a set constituted by few vertices to define a virtual backbone supporting information interchange is a problem that arises in many areas when analysing networks of different natures, like wireless, brain, or social networks. Recent papers propose obtaining such a set of vertices by computing the connected dominating set (CDS) of a graph. In recent works, the CDS has been obtained by considering that all vertices exhibit similar characteristics. However, that assumption is not valid for complex networks in which their vertices can play different roles. Therefore, we propose finding the CDS by taking into account several metrics which measure the importance of each network vertex e.g., error probability, entropy, or entropy variation (EV).

## 1. Introduction

Complex networks are playing an increasingly important role in a large number of areas (for instance, biology, physics, Social Science, etc.) [1,2]. The identification of the most relevant set of vertices in such networks allows us to better control the spread of disease [3], design marketing strategies [4], optimize limited resource allocation [5], and so on. In particular, connected dominating sets (CDSs) are natural candidates for vertices to be used for information interchange in any kind of network. They can also be used as a virtual backbone infrastructure in ad hoc wireless networks [6,7,8,9].

A CDS is a subset of vertices constituting a connected induced subgraph such that every vertex in the network is either in the CDS or has a neighbour in it [8,9]. For the effectiveness of a virtual backbone, the underlying CDS must be small in size. Since the problem of finding a minimum-sized CDS has been shown to be non-deterministic polynomial-time hardness (NP-hard) [8], the design of approximation algorithms has become an important issue for the study of CDSs. Thus, for the last twenty years, many researchers have explored approximation algorithms distinct to that of Guha and Khuller [10]. The heuristics for obtaining the CDS can be divided into two groups: the first is focused on evolving a CDS by growing a small trivial CDS [10], and the second group strives to find disconnected independent sets of vertices which are joined through a minimum spanning tree or Steiner tree [6,11,12]. Our approach is based on the algorithm presented in [11], which constructs the CDS in three phases: firstly, the dominating sets are determined by iteratively identifying the maximum degree of vertices to discover the highest cover vertices; secondly, they are connected through a Steiner tree; and finally, the algorithm prunes this tree to form the CDS without redundant vertices.

In practice, it is natural to assume that the vertices of the graph have some positive weights. In the context of wireless ad hoc networks, these weights usually reflect residual energy or capabilities of a node for a specific task. Thus, the computation of a CDS with a minimum number of nodes, also referred to as a minimum CDS (MCDS), can be extended to a minimum weighted CDS (MWCDS), by means of incorporating weights into each node with the objective of finding the CDS that minimizes the cost function of the total weight. Experimental literature is mainly focused on benchmarking and applications of algorithms for (weighted) connected dominating set problems. Ambühl and Erlebach [13] were the first to design a constant factor approximation algorithm for the MWCDS on a unit disk graph (UDG). They divided the region in square partitions and used the topological characteristics of the UDG to determine the vertices that covered each partition. Later, Huang et al. [14] proposed a strategy to reduce the computational cost by partitioning the whole plane into squares, and forming them into blocks. The MWCDS for each block was computed first, and then combined together to find the MWCDS of the graph. More recently in [15] the authors proposed including a new condition to the MWCDS of a UDG: all vertices in the MWDS must be connected with *k* vertices to guarantee redundancy. All these algorithms were designed for the UDG without taking into account the condition of minimizing the dominant size.

Unlike previous approaches, our work is focused on finding an MWCDS for any graph, without considering the topological characteristics. In the first stage, a set of dominating sets (DSs) is constructed taking into account the weight and the degree, and they are subsequently connected. Since we are also interested in reducing the dominant size, we include a prune stage to reduce the number of vertices in the MWCDS.

In addition, we will explain the relationship between the MWCDS and some theoretical concepts of metrics like error probability, entropy, and entropy variation. In particular, the study of the entropy to measure the information in a graph is a relevant topic in many areas. For instance, Rashevsky [16] proposed the concept of graph entropy to study the relationships between the topological properties of graphs and the information content in an organism. Mowshowitz and Dehmer [17,18] contextualized various entropy-based measures proposed from Rashevsky’s work. In [19], Kajdanowic and Morzy presented several simulation results oriented towards examining the usefulness of the entropy concept for different graph models. Moreover, in a recent paper, Ai [20] introduced the concept of entropy variation as a measurement of the influence of each vertex in the graph. In this sense, our study is oriented towards finding an MWCDS considering that graph information.

This paper is organized as follows. Section 2 includes some definitions for the understanding of this work. Section 3 explains the computation of a CDS considering vertex importance metrics. Some results are shown in Section 4, and some concluding remarks are briefly made in Section 5.

## 2. Previous Definitions

A graph G=(V;E) consists of a set of vertices, known as a *vertex set* and denoted by *V*, and of a set of edges, called the *edge set* and denoted by *E*. The vertices correspond to the objects to be modelled, while the edges indicate some relationship between pairs of these objects. For instance, in the case of social networks, the individuals of the population and the friendships among them are respectively represented by vertices and edges.

In our settings, the graphs are usually undirected i.e., if *u* is directly connected to *v*, then also *v* is directly connected to *u*.

**Definition** **1** **(Connected Dominating Set (CDS))**. A subset D of V dominates in G if every vertex of V−D has at least one neighbour in D. A subset D of V is connected and dominates if D dominates and the subgraph induced by D is connected.

This definition states that the CDS is a subset of vertices such that any pair of vertices can be joined by a path in the network and any vertex in the network either belongs to the CDS (CDS vertex) or has a neighbour in the CDS (non-CDS vertex).

In the following, we focus on graphs whose vertices have positive weights. For example, in the context of wireless ad hoc networks, these weights usually reflect capabilities of a node for a specific task.

For this purpose, we assume that a function of vertex reliability, denoted as *f*, is given.

**Definition** **2** **(Graph** **Entropy).** *Let f:V→R be an arbitrary function representing the vertex reliability in a graph G. For a vertex v we define*

(1) $$p\left(v\right)=\frac{f(v)}{\sum_{v\in V}f\left(v\right)}.$$

*Since $\sum_{v\in V}p\left(v\right)=1$, p(v) can be interpreted as a probability mass function so that the entropy of a graph G can be defined as follows*

(2) $$\begin{array}{cc} I_{f}\left(G\right) & =-\sum_{v\in V}p\left(v\right)\log_{2}\left(p\left(v\right)\right)=-\sum_{v\in V}\frac{f(v)}{\sum_{v\in V}f\left(v\right)}\log_{2}\left(\frac{f(v)}{\sum_{v\in V}f\left(v\right)}\right)\\ & =\log_{2}\left(\sum_{v\in V}f\left(v\right)\right)-\sum_{v\in V}\frac{f(v)}{\sum_{v\in V}f\left(v\right)}\log_{2}f\left(v\right). \end{array}$$

This definition corresponds to the concept of entropy of a discrete random variable introduced by Shannon in [21]. In [20], the entropy defined as in Equation (2) is interpreted as a measure of the amount of information encoded in the network structure, although it is not used as a metric of the vertex influence, also referred to as *vertex importance*. Thus, the entropy variation is introduced by the authors in [20] to give an idea of such an influence.

**Definition** **3** **(Entropy** **Variation** **(EV)).** *For a reliability function f, the entropy variation produced by removing the vertex v is defined by*

(3) $$\begin{array}{ccc} EV(v) & = & I_{f}\left(G\right)-I_{f}\left(G_{v}\right), \end{array}$$

*where $G_{v}=\left(V^{\prime },E^{\prime }\right)$ denotes the subgraph of G with a vertex set given by $V^{\prime }=V-\left\{v\right\}$ and whose edge set $E^{\prime }$ verifies that $e=\left\{u,w\right\}\in E^{\prime }$ if and only if u≠v and w≠v.*

## 3. CDS Computation Based on Vertex Importance

Taking into account that in real networks the vertices represent objects with different characteristics, we propose finding a CDS of a graph G=(V;E) satisfying two conditions: (1) the CDS must have few vertices; and (2) the CDS must maximize a metric related to the vertex importance. We will consider a general importance function which computes the importance of any vertex in the network according to some metrics and present several examples, including the entropy variation.

In general, for a fixed reliability function, *f*, and its associated probability function, *p*, we consider that an importance function is any function from *V* to R in a graph G=(V;E). We will denote it by *T*. For each vertex v∈V, the real number T(v) denotes the importance of the vertex *v*.

For a fixed reliability function *f* in a graph G=(V;E) and any importance function *T*, we denote *T*-CDS as the CDS verifying the following conditions,

- firstly, there is no other CDS of the *G* with a lower number of vertices;
- secondly, given several CDSs with the same number of vertices, the *T*-CDS maximizes a cost function given by
  (4) $$\begin{array}{ccc} J(D) & = & \sum_{v\in D}T\left(v\right), \end{array}$$
  where *D* is the set of vertices of the CDS.

For some graphs with a regular structure, such as those briefly described in the following examples, it is possible to propose a simple procedure that guarantees the computation of the optimum *T*-CDS. However, the computation of an MCDS for a random graph is an NP-hard problem [10] and the algorithms proposed in the literature give only a CDS with a reduced number of nodes without guaranteeing the condition of minimum size. As a consequence, the computation of the *T*-CDS is also an NP-hard problem. Section 3.2 presents the generalized algorithm proposed in this paper for the construction of a *T*-CDS in a suboptimal way.

**Example** **1** **(Bipartite** **Graph).** Let G be a bipartite graph of N vertices where each vertex is defined by an importance function T. The CDS with minimum size is formed by two vertices connected by an edge. Computing the value $t_{uv}=T\left(u\right)+T\left(v\right)$ for each pair of connected vertices u and v, the cost function J for any transformation T is maximized when the T-CDS consists of vertices u and v with maximum value of $t_{uv}$.

**Example** **2** **(Cycle** **Graph).** Let G be a cycle graph of N vertices where each vertex is defined by an importance function T. The CDS with minimum size is formed by a set of N−2 connected vertices. Computing the value $t_{uv}=T\left(u\right)+T\left(v\right)$ for each pair of connected vertices u and v, the cost function J for any transformation T is maximized when the T-CDS is formed by all the vertices excluding those u and v vertices with minimum values of $t_{uv}$.

### 3.1. Selection of the Importance Function

The *T*-CDS defined above can be particularized using any importance function *T*. In particular, we will consider the following vertex importance metrics given by the definitions

(5) $$\begin{array}{ccc} T_{1}\left(v\right) & = & \log_{2}p\left(v\right), \end{array}$$

(6) $$\begin{array}{ccc} T_{2}\left(v\right) & = & -p\left(v\right)\log_{2}\left(p\left(v\right)\right), \end{array}$$

(7) $$\begin{array}{ccc} T_{3}\left(v\right) & = & EV\left(v\right)=I_{f}\left(G\right)-I_{f}\left(G_{v}\right). \end{array}$$

**Example** **3.** *Given a graph G=(V;E) and the importance function $T_{1}\left(v\right)=\log_{2}p\left(v\right)$, we denote $T_{1}$-CDS as the CDS maximizing the following cost function:*

(8) $$J\left(D\right)=\sum_{v\in D}\log_{2}p\left(v\right).$$

Note that this function is related to the concept of error probability. Considering the vertices in the CDS i.e., v∈D, the error probability associated to the transmitted message through those vertices with such importances is given by

(9) $$P_{E}\left(D\right)=1-\prod_{v\in D}p\left(v\right).$$

Note that

(10) $$\min P_{E}\left(D\right)\equiv \max\prod_{v\in D}p\left(v\right)\equiv \max\log_{2}\left(\prod_{v\in D}p\left(v\right)\right)\equiv \max\sum_{v\in D}\log_{2}p\left(v\right).$$

**Example** **4.** *Given a graph G=(V;E) and the importance function $T_{2}\left(v\right)=-p\left(v\right)\log_{2}p\left(v\right)$, we denote $T_{2}$-CDS as the CDS maximizing the following cost function (entropy),*

(11) $$J\left(D\right)=-\sum_{v\in D}p\left(v\right)\log_{2}p\left(v\right).$$

**Example** **5.** *Given a graph G=(V;E) and the importance function $T_{3}\left(v\right)=EV\left(v\right)=I_{f}\left(G\right)-I_{f}\left(G_{v}\right)$, we denote $T_{3}$-CDS as the CDS maximizing the following cost function,*

(12) $$J\left(D\right)=\sum_{v\in D}\left(I_{f}\left(G\right)-I_{f}\left(G_{v}\right)\right),$$

*where $I_{f}\left(G\right)-I_{f}\left(G_{v}\right)$ is the entropy variation defined in Equation (3).*

### 3.2. Algorithm

Since the computation of an MCDS is an NP-hard problem [11], several approaches have been proposed in the literature [6,7,11]. In particular, the suboptimal algorithm proposed in [11] consists of three phases: the first one computes a disconnected DS; the second one connects the different DS subgraphs to build an initial CDS; and finally, the third phase prunes the resulting CDS so that the number of vertices is minimized. The problem presented by us in this paper is more complex because the optimum solutions would require the computation of all possible MCDSs and selection of the best according to the metric to be maximized. To reduce the computational cost we propose an algorithm which includes the metric at each step in order to determine the node to include or prune when there are several alternatives.

**Figure 1:**
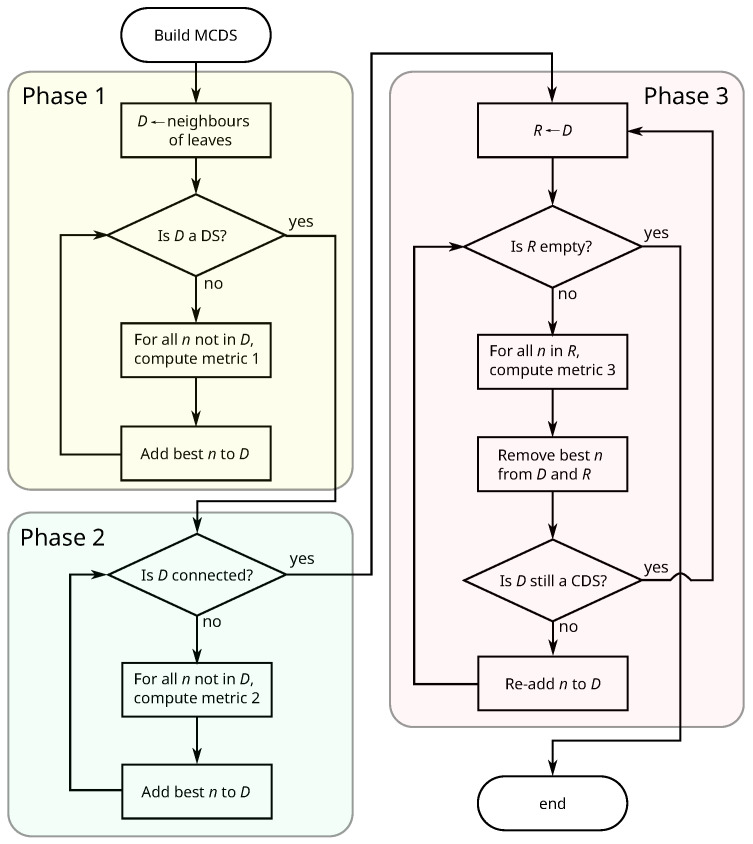
Flowchart for the computation of the minimum connected dominating set (MCDS).

Figure 1 shows a flowchart of the proposed algorithm. Each of the three phases uses a different metric to find the node to add to or remove from the CDS. The following sections explain each phase in detail.

#### 3.2.1. Phase 1: Find the Disconnected DS

The DS is initialized with the nodes that are neighbours of leaf nodes, since they must be in the final CDS. Then it is constructed by adding vertices until all of them are either part of the DS or neighbours of it. The vertices are chosen in order of highest value for the metric 1 indicated in the flowchart. We define this metric for each vertex as the number of vertices covered by the DS together with the vertex. In case there are more than one vertex with the same number, the vertex with the highest value of the importance function will be chosen.

The pseudo-code of the algorithm implemented for this first phase is detailed in Algorithm 1. In this pseudo-code, note that

(13) $$\mathrm{ARGMAX}\left(A,C\right)=\underset{n\in A}{\arg\;\max}\;C\left(n\right).$$

**Algorithm 1** Algorithm for phase 1: Disconnected DS.1:**procedure** Phase 1(*G*)2:  *DS* ← neighbors(leaves(*G*))3:  **while** *DS* ∪ neighbors(*DS*) ≠ *G* **do**4:    R←G−DS5:    **for all** vertex∈R **do**6:      $DS^{\prime }\leftarrow DS\cup \left\{vertex\right\}$7:      N(vertex)← size(*DS*′ ∪ neighbors(*DS*′))8:    **end for**9:    L1← argmax(*R, N*)10:    vertex← first(argmax(*L*1, J))11:    DS←DS∪{vertex}12:  **end while**13:**end procedure**


#### 3.2.2. Phase 2: Compute Initial CDS

The CDS is constructed by adding vertices which connect the different disconnected subgraphs of the DS resulting from the previous phase. The order in which they are added (metric 2 in the flowchart) is: first, those that connect a higher number of disconnected DS subgraphs; then, in the case of several vertices connecting the same number of subgraphs, those with the highest degree; and finally, if there are multiple vertices of equal degree, that with the highest value of the importance function.

In this phase we need to make use of an auxiliary algorithm for finding the number of disconnected subgraphs, as shown in Algorithm 2.

**Algorithm 2** Auxiliary algorithm for phase 2.
1:**function** n_subgraphs(*G*)2:  n←03:  R←G4:  **while** R≠∅ **do**5:    S← {first(*R*)}6:    **while** S≠S ∪ neighbors(*S*) **do**7:      S←S ∪ neighbors(*S*)8:    **end while**9:    R←R−S10:    n←n+111:  **end while**12:  **return** *n*13:**end function**


The pseudo-code of the algorithm used for connecting the CDS is shown in Algorithm 3. Again, note that

(14) $$\mathrm{ARGMIN}\left(A,C\right)=\underset{n\in A}{\arg\;\min}\;C\left(n\right).$$

**Algorithm 3** Algorithm for phase 2: CDS.1:**procedure** Phase 2(*G*, DS)2:  CDS←DS3:  R←G−CDS4:  **while** is_disconnected(*CDS*) **do**5:    **for all** vertex∈R **do**6:      N(vertex)← n_subgraphs(*CDS* ∪ {*vertex*})7:    **end for**8:    L1← argmin(*R, N*)9:    L2← argmax(*L*1, degree)10:    vertex← first(argmax(*L*2, J))11:    CDS←CDS∪{vertex}12:    R←R−{vertex}13:  **end while**14:**end procedure**


#### 3.2.3. Phase 3: Prune Vertices

The final CDS results from pruning the CDS obtained from the previous phase, since there can be vertices added in the first phase that are no longer necessary after the second phase. The vertices are removed according to metric 3 in order of the lowest degree; in the case of several vertices with the same degree, those with lowest degree to nodes in the CDS are chosen. If there are several such nodes, that with a lowest value of the importance function is chosen. Note that every time a vertex is pruned the process must be restarted.

The pseudo-code of the algorithm used for this third phase is shown in Algorithm 4.

**Algorithm 4** Algorithm for phase 3: Pruning.1:**procedure** Phase 3(G,CDS)2:  R←CDS3:  **while** R≠∅ **do**4:    L1← argmin(*R*, degree)5:    L2← argmin(*L*1, degree(*CDS*))6:    vertex← first(argmin(*L*2, J))7:    TCDS←CDS−{vertex}8:    **if** connected (TCDS) & TCDS ∪ neighbors(*TCDS*) = *G* **then**9:      CDS←TCDS10:      Phase 3(G,CDS)11:    **else**12:      R←R−{vertex}13:    **end if**14:  **end while**15:**end procedure**


## 4. Results and Discussion

In this section we perform several simulations to verify that the proposed algorithm allows us to compute a CDS formed by a reduced number of vertices with a good performance in terms of maximization of importance functions.

In the literature, we can find several theoretical graph models proposed to construct graphs that would display certain properties frequently appearing in empirical graphs (see, for instance the review in [19]). In particular, we will consider the UDG [8] and the small-world model [19].

### 4.1. Unit Disk Graph

We have considered an ad hoc wireless network which is a decentralized type of wireless network characterized by a lack of fixed communication infrastructure, so that the selection of vertices forwarding data is dynamically made by considering the current network connectivity. Several researchers have proposed using the CDS as a virtual backbone in these networks as an alternative to the fixed routing infrastructure in classical wired networks [6,7]. The virtual backbone represents the "skeleton” of the entire network and is used to frequently exchange routing information (traffic conditions, neighbourhood information, etc.) and broadcast a message in the network.

**Figure 2:**
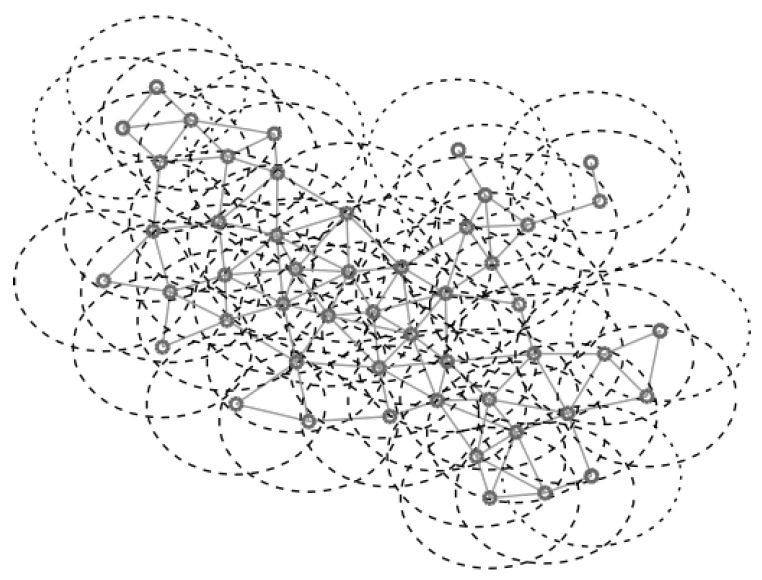
Example of a unit disk graph (UDG).

**Figure 3:**
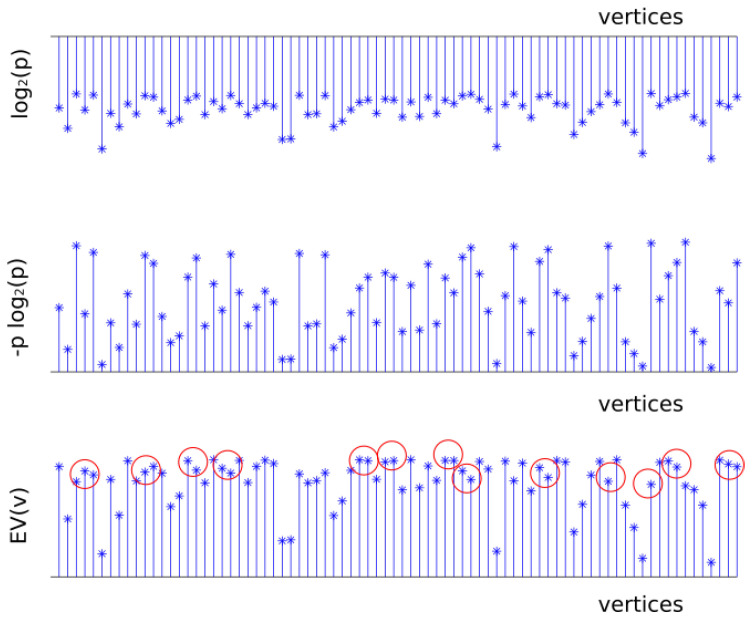
Importance functions *T*. It is interesting to observe that $T_{1}$ and $T_{2}$ have the same trend but $T_{3}$ presents some differences which are marked in red.

For the UDG model the network is defined by G=(V;E), where the vertices in *V* are embedded in the Euclidean plane. We assume that the maximum transmission range is the same for all the vertices in the network and it is unit scaled. There exists an edge {u,v}∈E if *u* and *v* are in the maximum transmission range of each other i.e., the Euclidean distance is d(u,v)≤1. Figure 2 shows an example of a UDG of 50 vertices with the coverage radius of each vertex. Figure 3 shows the values of $T_{1}\left(v\right)=\log_{2}\left(p\left(v\right)\right)$, $T_{2}\left(v\right)=-p\left(v\right)\log_{2}\left(p\left(v\right)\right)$, and $T_{3}\left(v\right)=EV\left(v\right)$, obtained generating *f* according to a uniform distribution in the interval (0,1]. It is interesting to observe that $T_{1}\left(v\right)=\log_{2}\left(p\left(v\right)\right)$ and $T_{2}\left(v\right)=-p\left(v\right)\log_{2}\left(p\left(v\right)\right)$ have the same trend but $T_{3}\left(v\right)=EV\left(v\right)$ presents some differences which are marked in red in the figure.

**Figure 4:**
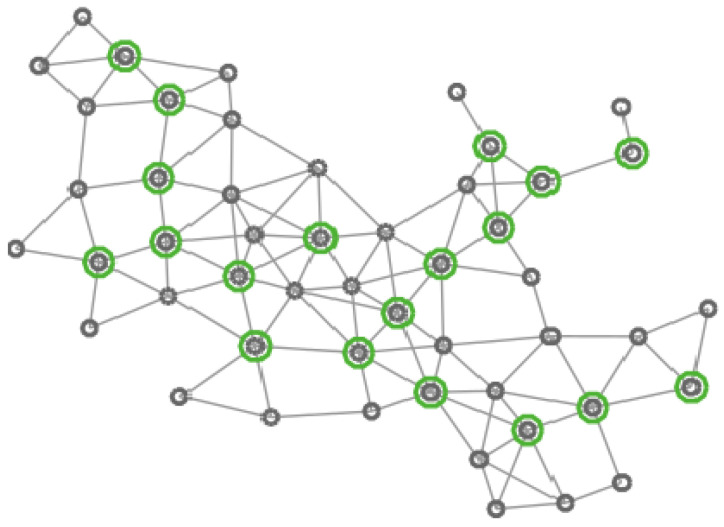
Graph of 50 vertices. Vertices in the 1-CDS are marked in green.

**Figure 5:**
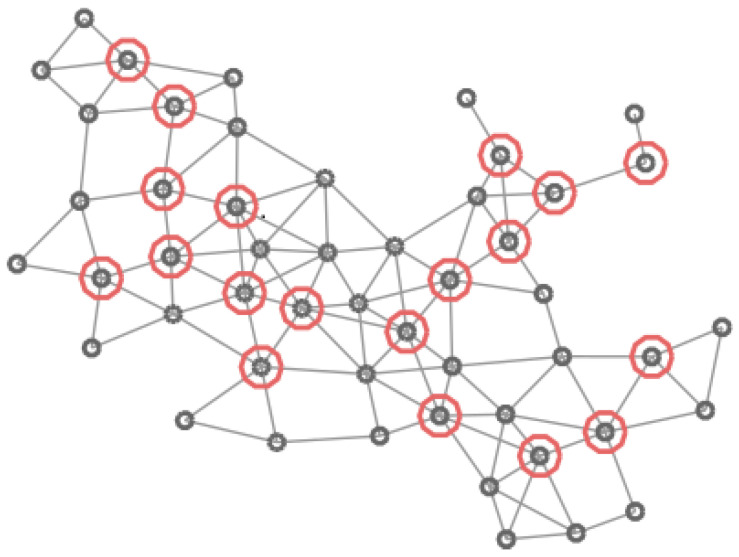
Graph of 50 vertices. Vertices in the $T_{1}$-CDS are marked in red.

**Figure 6:**
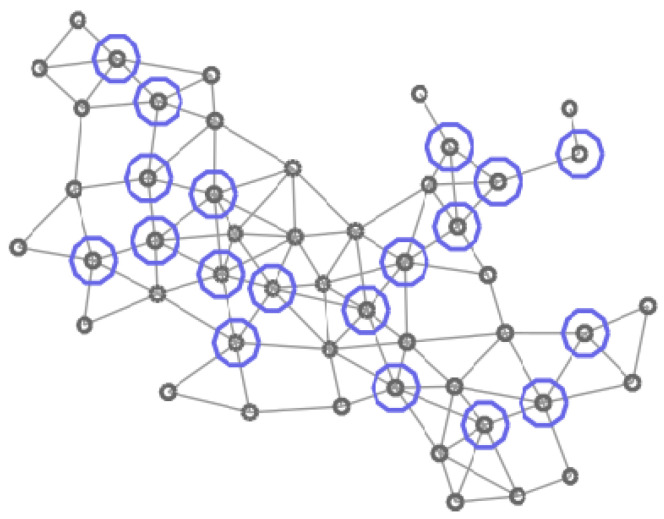
Graph of 50 vertices. Vertices in the $T_{2}$-CDS are marked in blue.

**Figure 7:**
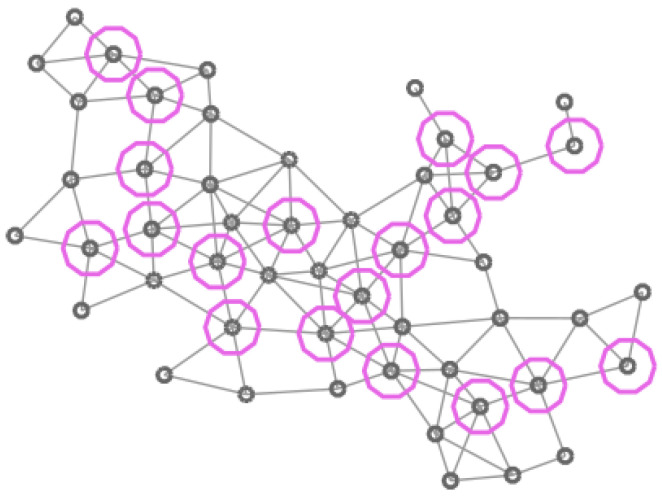
Graph of 50 vertices. Vertices in the $T_{3}$-CDS are marked in pink.

In Figure 4, Figure 5, Figure 6 and Figure 7 four different CDS can be observed: a CDS without using a vertex importance metric (called 1-CDS), the $T_{1}$-CDS, the $T_{2}$-CDS, and the $T_{3}$-CDS. Note that there are variations between the four configurations although all of them are constituted by the same number of vertices (19 out of initial 50 vertices).

We have considered graphs with 20, 50, and 80 vertices with randomly generated connections. The function *f* of each vertex follows a standard uniform distribution. We have generated 1000 realizations of different graphs for each one of those sizes. The CDS corresponding to each approach above depicted is computed so that its size is minimal and in the case of vertex importance metrics, the respective cost function given by Equations (8), (11), or (12), must be maximized for the obtained CDS. For all these CDSs, we have calculated the maximum value of every importance function, denoted by *b*, and its deviation with respect to that maximum so that we can obtain the parameter

(15) $$\gamma =\frac{{\left(r-b\right)}^{2}}{b^{2}},$$

where *r* is the value of the importance function obtained by any CDS.

Table 1 shows the number of times, expressed as a percentage, that the four CDS achieve the minimum size. Data shown in the table demonstrate that all the CDS are similar in terms of size. This table also shows the mean deviation obtained by averaging the results of evaluating Equation (15) throughout 1000 realizations, with *b* being the minimum value of the four CDS sizes. This deviation is very small because of difference in size is less than two vertices for all the network sizes. Note also that $T_{1}$-CDS and $T_{2}$-CDS give exactly same results.

**Figure 8:**
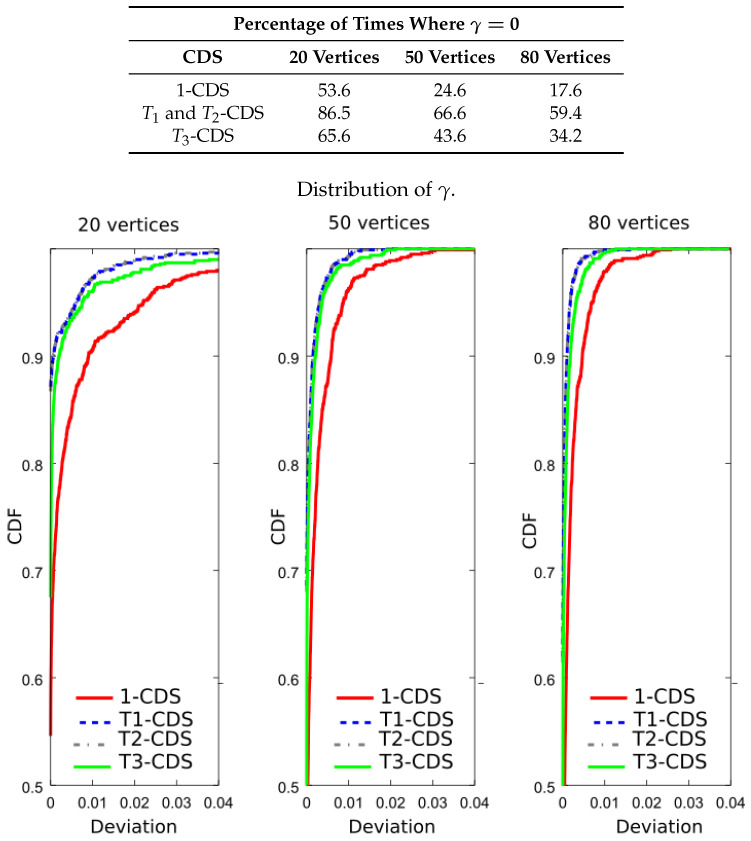
Analysis of the UDG: percentage of times where γ=0 and cumulative distribution function (CDF) of γ for a metric based on the error probability, $J\left(D\right)=\sum_{v\in D}\log_{2}\left(p\left(v\right)\right)$.

Now, we wish to verify that $T_{1}$-CDS reduces the error probability. For this purpose, we evaluated the importance function shown in Equation (8) for the four CDSs. The table included in Figure 8 shows the result percentages in terms of number of times the maximum value of the importance function is achieved, i.e., γ=0. We see that $T_{1}$-CDS and $T_{2}$-CDS exhibit the same performance, at 86.5% for 20 vertices. The difference with respect to 1-CDS and $T_{3}$-CDS is also remarkable for 50 and 80 vertices. Figure 8 also depicts the cumulative distribution function (CDF) of the function γ given in Equation (15) (curves of $T_{1}$-CDS and $T_{2}$-CDS are represented in the same line). We observe that the difference appears depending on the applied method reduces with the graph size. Note that 1-CDS shows poor performance since the number of times it achieves the maximum value of the importance function is lower than that exhibited by the other methods.

**Figure 9:**
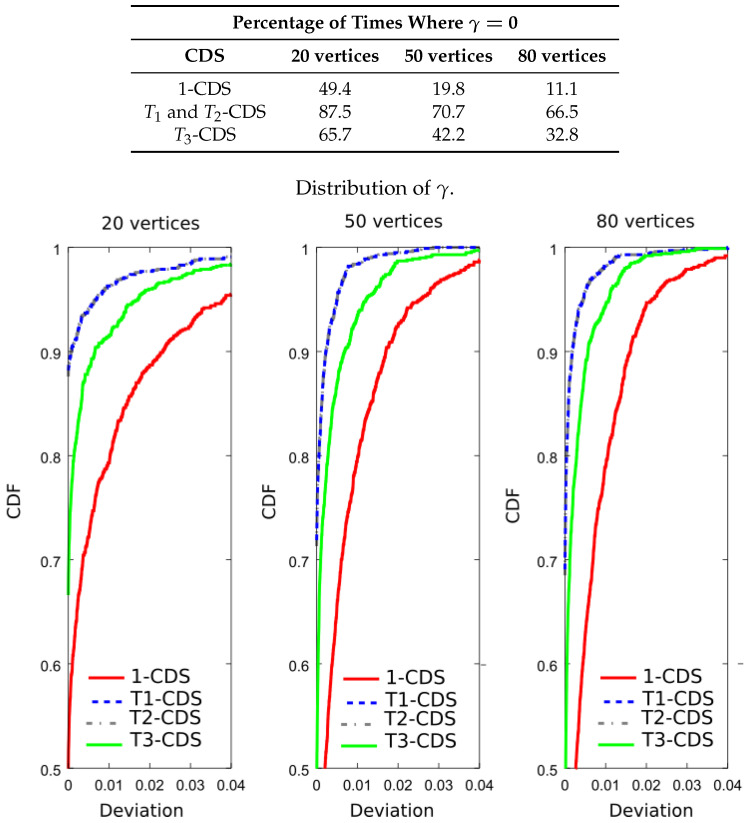
Analysis of the UDG: percentage of times where γ=0 and CDF of γ for a metric based on the entropy, $J\left(D\right)=-\sum_{v\in D}p\left(v\right)\log_{2}\left(p\left(v\right)\right)$.

Following the computer experiments, we compared the entropy of the computed CDS. For that purpose, we have evaluated Equation (11) to calculate the percentage of times in which each CDS achieves the maximum value of the importance function. The table included in Figure 9 shows the new results. It is apparent that $T_{1}$-CDS and $T_{2}$-CDS achieve the best performances with a considerable gap with respect to the rest of the algorithms. The same observation can be made if we see the CDF in Figure 9: $T_{1}$-CDS and $T_{2}$-CDS have a high probability regardless of the network size, while the other methods present a considerable error. Again, 1-CDS provides the worst results.

**Figure 10:**
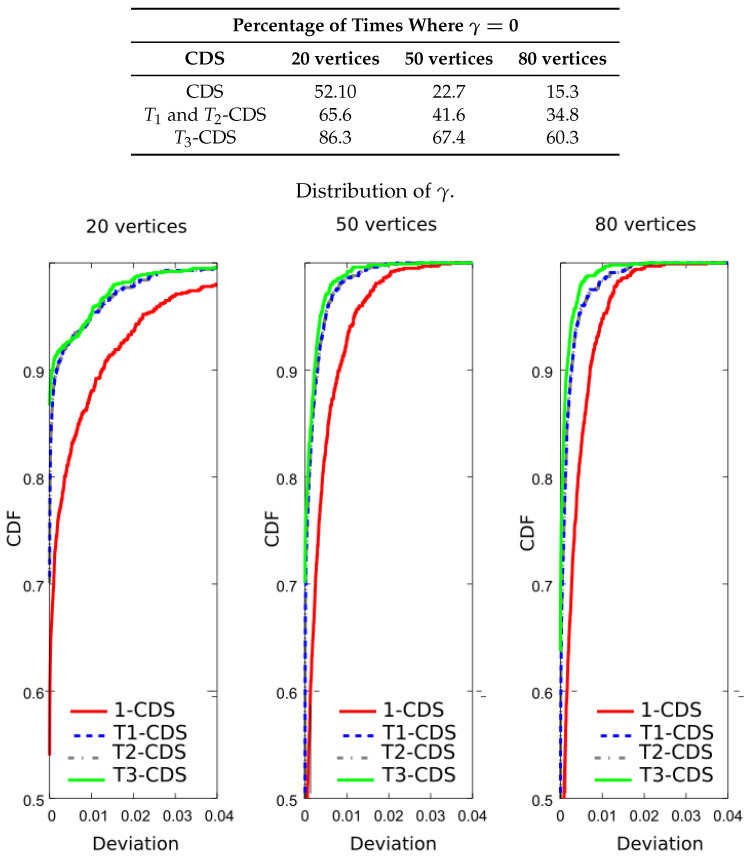
Analysis of the UDG: percentage of times where γ=0 and CDF of γ for a metric based on the sum of entropy variation, $J\left(D\right)=\sum_{v\in D}EV\left(v\right)$.

Finally, we compared the CDS in terms of the sum of entropy variation. We evaluated Equation (12) for the vertices of the obtained CDS. Figure 10 shows that $T_{3}$-CDS gives the best performances in terms of the percentages above explained although the differences in CDF compared to the $T_{1}$-CDS and $T_{2}$-CDS are negligible.

Therefore, it can be said that the algorithms proposed in this paper are correctly working in the sense of maximizing their cost function using a reduced number of vertices, and that the metrics defined in Equations (8) and (11) show same performances, while the metric of entropy variation (see Equation (12)) presents differences that we will try to analyse. 1-CDS provides the worst results for all the defined metrics since the algorithm only considers the vertex degree.

### 4.2. Small-World Model

The small-world model was introduced in [22]. According with this model, a set of *N* vertices is organized into a regular circular graph where each vertex is directly connected to a mean number of *K*-nearest neighbours. For each edge in the graph, the target node with probability β is rewired. When β=1, the small world graph becomes the random graph. In our simulations, we generated 1000 realizations of graphs with 20,50, and 80 vertices with β=0.5 and K=N/2 (i.e, a mean number of 10, 25, and 40 connections for any vertex). The function *f* follows a uniform distribution in the interval (0,1].

Table 2 shows the number of times, expressed as a percentage, where the CDS achieved the minimum size. We can see that, as occurs in the UDG, there is no a remarkable difference between the four CDSs. However, the deviation with respect to the optimum value is considerably higher than for the UDG graph. This means that in those occasions where the CDS does not achieve the best size, the CDS size differs in more than two vertices from the optimum.

We evaluated the deviation of Equation (15) in order to verify the correct behavior of the proposed algorithm. Table 3, Table 4 and Table 5 show the result percentages in terms of number of times achieving the maximum value of the importance functions in Equations (8), (11), and (12), respectively. We can see that each *T*-CDS gives the best result for the corresponding importance metric. In general, the results are similar to those obtained with UDG.

### 4.3. Importance Function Comparison

In order to compare the three importance functions, we will consider a graph formed by *N* nodes, denoted by $v_{i}$, with $f\left(v_{i}\right)=f_{i}=i/N$, where i=1,2,…,N. Figure 8 shows the three importance functions for N=20, 50, and 80. From the top figure, we can observe that the $T_{1}\left(v_{i}\right)=\log_{2}\left(p\left(v_{i}\right)\right)$ function is increasing with respect to *f*. In fact, for our discrete distribution, we can find the analytical expression

(16) $$\begin{array}{cc} T_{1}\left(v_{i}\right) & =\log_{2}\left(p_{i}\right)=\log_{2}\left(f_{i}\right)-\log_{2}\left(\sum_{k=1}^{N}f_{k}\right)\\ & =\log_{2}\left(\frac{i}{N}\right)-\log_{2}\left(\sum_{k=1}^{N}\frac{k}{N}\right)=\log_{2}\left(\frac{i}{N}\right)-\log_{2}\left(\frac{N+1}{2}\right). \end{array}$$

**Figure 11:**
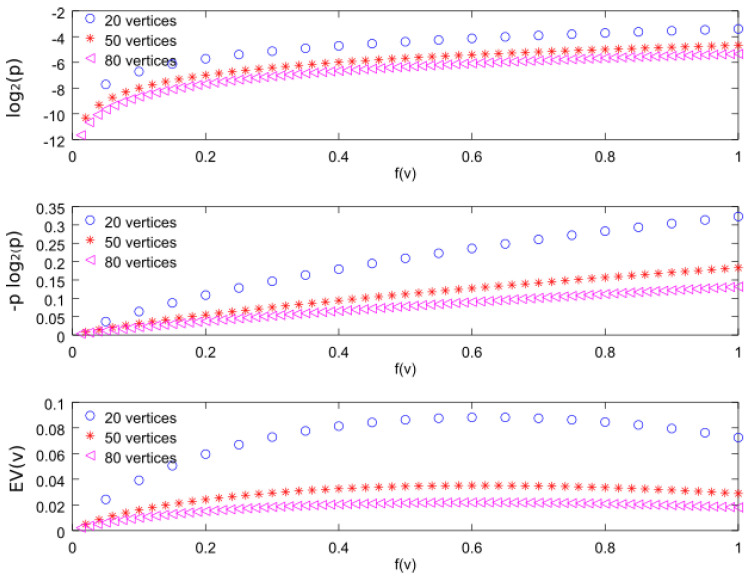
Importance functions for values of f(v)=i/N with i=1,2,…,N and N=20,50, and 80.

In Figure 11, we can see that the curves converge for large number of vertices (N=50 and N=80).

The second importance function represented in the figure is $T_{2}\left(v_{i}\right)=-p\left(v_{i}\right)\log_{2}\left(p\left(v_{i}\right)\right)$. This metric allows us to maximize the entropy. The analytical expression for $f\left(v_{i}\right)=f_{i}=i/N$ is given by

(17) $$\begin{array}{cc} T_{2}\left(v_{i}\right) & =-p_{i}\log_{2}\left(p_{i}\right)=-\frac{f_{i}}{\sum_{k=1}^{N}f_{k}}\left(\log_{2}\left(f_{i}\right)-\log_{2}\left(\sum_{k=1}^{N}f_{k}\right)\right)\\ & =\frac{2i}{N^{2}+N}\left(\log_{2}\left(\frac{N+1}{2}\right)-\log_{2}\left(\frac{i}{N}\right)\right). \end{array}$$

Note that the $N^{2}$ term has an important influence on the curve values, as it can be seen in Figure 11, but again the curves converge for large number of vertices. The importance function $T_{2}\left(v_{i}\right)=-p\left(v_{i}\right)\log_{2}\left(p\left(v_{i}\right)\right)$ increases with respect to *f*, as happens with $T_{1}\left(v_{i}\right)=\log_{2}\left(p\left(v_{i}\right)\right)$, and, for this reason, $T_{1}$-CDS and $T_{2}$-CDS give the same results in the simulation figure above presented.

Finally, the bottom figure represents the third importance function considered in this work i.e., $T_{3}\left(v_{i}\right)=EV\left(v_{i}\right)$. We can observe that it is an increasing function with *f*, similarly to the first two functions, although for higher *f* it decreases with a smaller slope. By evaluating Equation (2) for $f\left(v_{i}\right)=f_{i}=i/N$, we can express this importance function as follows:

(18) $$\begin{array}{cc} T_{3}\left(v_{i}\right) & =I_{f}\left(G\right)-I_{f}\left(G_{v_{i}}\right)\\ & =I_{f}\left(G\right)-\log_{2}\left(\frac{N+1}{2}-\frac{i}{N}\right)+\frac{1}{\frac{N+1}{2}-\frac{i}{N}}\left(\sum_{k=1}^{N}\frac{k}{N}\log_{2}\left(\frac{k}{N}\right)-\frac{i}{N}\log_{2}\left(\frac{i}{N}\right)\right)\\ & =I_{f}\left(G\right)-\log_{2}\left(\frac{N^{2}+N-2i}{2N}\right)+\frac{2N}{N^{2}+N-2i}\left(\sum_{k=1}^{N}\frac{k}{N}\log_{2}\left(\frac{k}{N}\right)-\frac{i}{N}\log_{2}\left(\frac{i}{N}\right)\right), \end{array}$$

where $I_{f}\left(G\right)$ is constant for a given graph. As can be seen in Figure 11, we can directly observe that the maximum values are close to 0.60 regardless of the vertex number.

Using simulations we confirmed that the value 0.60 obtained for $f\left(v_{i}\right)=f_{i}=i/N$ is also valid for random samples of a uniform distribution. For that, we generated 1000 samples of a uniform distribution and computed EV(v) using Equation (12). We found that the maximum values for N=20, 50, and 80 vertices are, respectively, 0.6055, 0.6023, and 0.6059.

Therefore, we can conclude that the importance function EV(v) behaves in a similar way to the other two when f(v)<0.60. For this reason, the $T_{3}$-CDS does not maximize the error probability or the entropy.

## 5. Conclusions

In this paper we proposed selecting the CDS of a graph by incorporating vertex importance metrics defined in order to maximize a desired cost function such as error probability, entropy, or entropy variation. We have shown that finding the optimum CDS is very simple for the bipartite graph and the cycle graph. For the general case, the computation of such a CDS is an 1/N-hard problem and we proposed an algorithm which selects the vertices in the CDS taking into account the defined importance metric and the vertex degree. Several simulation results show that the proposed algorithm allows us to find a CDS formed by a reduced number of nodes, similarly to previous methods, with the advantage of maximizing the objective metric. 

## Figures and Tables

**Table 1:** Size of the CDSs for the UDG.

Graph Size						
CDS	20 Vertices		50 Vertices		80 Vertices	
	Percentage	Mean Deviation	Percentage	Mean Deviation	Percentage	Mean Deviation
1-CDS	95	0.0007	82	0.0005	70	0.0005
$T_{1}$ and $T_{2}$-CDS	92	0.0012	78	0.0006	67	0.0005
$T_{3}$-CDS	92	0.0012	79	0.0006	65	0.0006

**Table 2:** Size of CDS for the small-world model.

Graph Size						
CDS	20 Vertices		50 Vertices		80 Vertices	
	Perc.	Mean Deviation	Perc.	Mean Deviation	Perc.	Mean Deviation
1-CDS	94	0.013	85	0.016	88	0.009
$T_{1}$ and $T_{2}$-CDS	92	0.016	86	0.014	88	0.008
$T_{3}$-CDS	94	0.010	85	0.017	87	0.010

**Table 3:** Analysis of the small-world model for a metric based on the error probability, $J\left(D\right)=\sum_{v\in D}\log_{2}\left(p\left(v\right)\right)$.

Percentage of Times Where γ=0			
CDS	20 Vertices	50 Vertices	80 Vertices
1-CDS	53.1	44.5	45.0
$T_{1}$ and $T_{2}$-CDS	82.0	73.9	73.9
$T_{3}$-CDS	64.3	55.2	51.5

**Table 4:** Analysis of the small-world model for a metric based on the entropy, $J\left(D\right)=-\sum_{v\in D}p\left(v\right)\log_{2}\left(p\left(v\right)\right)$.

Percentage of Times Where γ=0			
CDS	20 Vertices	50 Vertices	80 Vertices
1-CDS	51.7	42.3	41.5
$T_{1}$ and $T_{2}$-CDS	84.6	75.3	76.7
$T_{3}$-CDS	62.7	54.8	50.5

**Table 5:** Analysis of the small-world model for a metric based on the sum of entropy variation, $J\left(D\right)=\sum_{v\in D}EV\left(v\right)$.

Percentage of Times Where γ=0			
CDS	20 Vertices	50 Vertices	80 Vertices
CDS	52.3	44.0	43.7
$T_{1}$ and $T_{2}$-CDS	65.7	52.4	48.6
$T_{3}$-CDS	80.6	75.0	74.4

## Abbreviations

The following abbreviations are used in this manuscript:

CDS	connected dominating set
CDF	cumulative distribution function
DS	dominating set
EV	entropy variation
MCDS	minimum connected dominating set
MWCDS	minimum weighted connected dominating set
NP-hard	non-deterministic polynomial-time hardness
UDG	unit disk graph
